# A Review on Bioactive Compounds from Marine-Derived *Chaetomium* Species

**DOI:** 10.4014/jmb.2201.01007

**Published:** 2022-05-04

**Authors:** Yuan Tian, Yanling Li

**Affiliations:** College of Life Science, Shandong First Medical University & Shandong Academy of Medical Sciences, Taian 271016, P.R. China

**Keywords:** *Chaetomium*, secondary metabolite, structural diversity, biological activity

## Abstract

Filamentous marine fungi have proven to be a plentiful source of new natural products. *Chaetomium*, a widely distributed fungal genus in the marine environment, has gained much interest within the scientific community. In the last 20 years, many potential secondary metabolites have been detected from marine-derived *Chaetomium*. In this review, we attempt to provide a comprehensive summary of the natural products produced by marine-derived *Chaetomium* species. A total of 122 secondary metabolites that were described from 2001 to 2021 are covered. The structural diversity of the compounds, along with details of the sources and relevant biological properties are also provided, while the relationships between structures and their bioactivities are discussed. It is our expectation that this review will be of benefit to drug development and innovation.

## Introduction

*Chaetomium* is a large genus in the Chaetomiaceae family of Ascomycota, which contains more than 100 species [1]. *Chaetomium* is widely distributed in soil, air, animals and plants. It is an important resource for its production of active natural products and its main types of metabolites include cytochalasans, indole alkaloids, terpenoids, steroids, flavonoids, azaphilones, and so on, and their biological functions include enzyme inhibitory, antitumor, antimalarial, antioxidant, antibacterial and other activities [2].

The progress of research into the secondary metabolites originating from the genus *Chaetomium* has attracted much attention, and a series of excellent reviews on this topic have so far been published [2-5]. In 2012, Zhang *et al*. reviewed the extraction, elucidation, structural diversity and biological activities of natural products from terrestrial and marine-derived *Chaetomium* between 1936 and 2010. The secondary products were categorized into 21 structural types and included chaetoglobosins, diketopiperazines, tetramic acids, isoquinolines, azaphilones, terpenoids, etc. Some bioactive compounds as well as their mechanisms of action and structure-activity relationships were highlighted in the literature [2]. In 2016, Nighat *et al*. reviewed the isolation of *Chaetomium* endophytes, the extraction and isolation of metabolites, and their biological activities. The bioactive molecules were classified into five types, including anticancer and cytotoxic metabolites, antimicrobial metabolites, enzyme inhibitors, antimalarial and antitrypanosomal metabolites, as well as antioxidant metabolites [3]. Non-endophytic *Chaetomium* species were not included in the literature. In 2018, two Chinese studies reported the progress of various research projects involving *Chaetomium*. Xu *et al*. summarized the information on 208 secondary metabolites from *Chaetomium* fungi, which had been reported from 2011 to 2016 [4]. Liang reviewed the diversities and bioactivites of the secondary metabolites from *Chaetomium globosum* between 2015 and 2017 [5]. However, no work has been speciﬁcally focused on *Chaetomium* species from the marine environment. Marine fungi are important sources of secondary metabolites for drug discovery [6], and moreover, reports of new natural products from marine-derived fungi have increased dramatically over the last few decades [7]. It is believed that the exploration of fungi living in the marine environment will advance the isolation of new fungal species and lead to the discovery of novel compounds [8].

Therefore, in this review, we describe the structural diversities and biological activities of 122 compounds isolated from marine-derived *Chaetomium* species over the past 20 years (2001 to 2021). The review also expounds the relationship between the structures and functions of the natural products, and improve understanding of the fascinating chemistry and bioactivity of the natural products resulting from marine *Chaetomium* species.

## Bioactive Compounds from Marine-Derived *Chaetomium* Species

Marine *Chaetomium*-derived compounds with various structures offer abundant bioactive core skeletons for new medicinal lead molecules. The different structural types of these compounds, including cytochalasans, dioxopiperazines, indole alkaloids, azaphilones, xanthone derivatives and others, are discussed below. The metabolites name index in combination with the structure type index, biological activity index and the references on isolation are listed in Table 1.

## Cytochalasans

Cytochalasans are a large group of fungal alkaloids with a wide range of biological activities. They have been an important chemical tool in cell and molecular biology. Some of them also possess phytotoxic, cytotoxic, and antibiotic activities [9, 10]. Cytochalasans are characterized by a highly substituted perhydro-isoindolone moiety incorporating a macrocyclic ring [11]. The fungal polyketide synthase nonribosomal peptide synthetase (PKS-NRPS) plays an important role in forming cytochalasans [12].

**Fig. 1:**
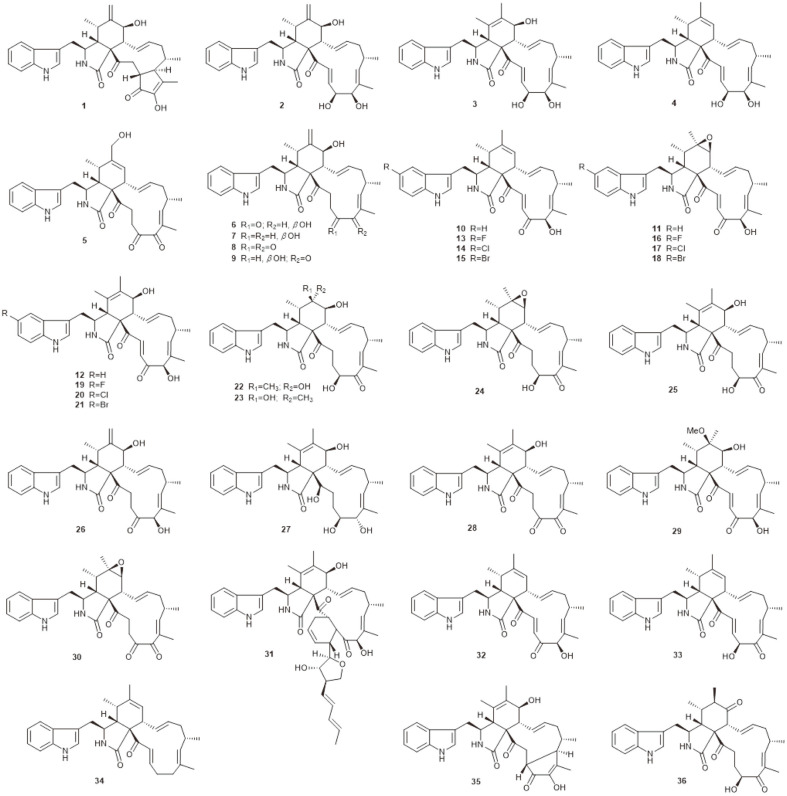
Cytochalasans produced by marine-derived *Chaetomium* species.

Cytoglobosins, chaetoglobosins and penochalasins are common classes of cytochalasan alkaloids in *Chaetomium*. Seven new cytochalasan derivatives cytoglobosins A–G (**1–7**), together with two known, structurally related compounds, isochaetoglobosin D (**8**) and chaetoglobosin F_ex_ (**9**) (Fig. 1), were isolated from *C. Globosum* QEN-14, an endophytic fungus derived from the marine green alga *Ulva pertusa*. Cytoglobosins C (**3**) and D (**4**) showed moderate activity against A-549 cell line with IC_50_ values of 2.26 and 2.55 μM, respectively [13]. From *C. globosum* 1C51, a fungus residing inside the gut of an ocean ﬁsh, *Epinephelus drummondhayi*, three metabolite chaetoglobosins, A, B and J (**10–12**) (Fig. 1), were obtained with high yields. Using precursor-directed biosynthesis, nine halogenated derivatives were produced (**13–21**) (Fig. 1). All isolated compounds were tested and found to be immunosuppressive, and compound **18** had the most potential with a high selectivity index (SI = 26.6) [14].

Chemical investigation of deep-sea-derived fungus *C. globosum* MCCC 3A00607 resulted in obtaining two new compounds, cytoglobosins H (**22**) and I (**23**), together with seven known ones: cytoglobosins B (**2**) and C (**3**), chaetoglobosins F_ex_ (**9**), F (**24**), E (**25**), and B (**11**), and isochaetoglobosin D (**8**) (Fig. 1). Compound **25** exhibited signiﬁcant antiproliferative activity on LNCaP human prostate cancer cells and B16F10 mouse melanoma cells with IC_50_ values of 0.62 and 2.78 μM, respectively [15]. Chemical investigation of the EtOAc extract of the gorgonian-derived fungus *C. globosum* RA07-3 resulted in the isolation of six cytochalasans, chaetoglobosins A and B (**10** and **11**), cytoglobosin C (**3**), chaetoglobosins D–F (**26, 25, 24**) (Fig. 1), together with three other compounds, xylariol A (**107**), (3R,4S)-6,8-dihydroxy-3,4,5-trimethylisochroman-1-one (**108**), and 2,5,8-benzotrioxacycloundecin-1,9-dione (**109**) (Fig. 7). Compounds **10** and **11** exhibited moderate brine shrimp lethality with LC_50_ values of 9.72 and 12.41 μg/mL, respectively. Compounds **10, 11** and **3** also exhibited strong antibacterial activities against *Tetragenococcus halophilus* with MIC values of 0.7, 0.4, and 0.7 μM, respectively [16].

Gene mining of the sea cucumber-associated fungus *C. globosum* led to the new cytoglobosin X (**27**) and the known cytochalasans chaetoglobosin F_ex_ (**9**), G (**28**), and B (**11**) (Fig. 1), together with a known indole alkaloid cochliodinol (**41**) (Fig. 3) and four known azaphilones, chaetomugilin A (**56**) (Fig. 4), and chaetoviridins A (**86**), E (**87**), and B (**100**) (Fig. 5). Compound **11** showed moderate activity against *Staphylococcus aureus* and methicillin-resistant *S. aureusi* (MRSA) with MIC values of 47.3 and 94.6 μM, respectively [17]. Recently, sixteen structurally diverse chaetoglobosins were isolated from the coral-associated fungus *C. globosum* C2F17, including a new one, 6-O-methyl-chaetoglobosin Q (**29**), along with the previously isolated chaetoglobosins A (**11**), B (**12**), C (**30**), D (**26**), E (**25**), F (**24**), G (**28**), and aureochaetoglobosin (**31**), isochaetoglobosin D (**8**), chaetoglobosin F_ex_ (**9**), penochalasin G (**32**), armochaetoglobin G (**33**), prochaetoglobosin I (**34**), chaetoglobosinV_b_ (**35**), and chaetoglobosin Y (**36**) (Fig. 1). Among them, compound 25 showed signiﬁcant cytotoxicity against K562, A549, Huh7, H1975, MCF-7, U937, BGC823, HL60, HeLa, and MOLT-4 cell lines, with IC_50_ values ranging from 1.4 to 9.2 μM. Additionally, compound 9 displayed selective cytotoxic activity against Huh7, MCF-7, U937 and MOLT-4 cell lines, with IC_50_ values of 3.0, 7.5, 4.9, and 2.9 μM, respectively [18].

A total of 36 cytochalasans were obtained from marine-derived *Chaetomium* species, among which, compounds **3, 4, 9**, and **25** showed cytotoxic activity. Several researchers have investigated the structure-activity relationships of cytochalasans and concluded that besides the intact macrocycle, the hydroxyl function at C-7 was an important pharmacophore concerning cytotoxic activity [19-21]. In this review, except compound **4**, all of the compounds with cytotoxicity have hydroxyl function at C-7. All of the halogenated cytochalasans (**13–21**) were found to be immunosuppressive, indicating that halogen atoms may be important functional groups that confer immunosuppressive activity. It has been reported that the presence of an α,β-unsaturated carbonyl group in the macrolide moiety was a prerequisite for activity against gram-positive bacteria [22]. Furthermore, the three bacteriostatic compounds, **3, 10**, and **11** all have the C-7 α,β-unsaturated carbonyl group in their structures. As more cytochalasans are increasingly being discovered, researchers are continually gaining new insights into the structure-activity relationship.

## Dioxopiperazines

Dioxopiperazines are common metabolites of microorganisms that are distributed in a diverse range of ﬁlamentous fungi [23, 24]. Many dioxtopiperazines reported from marine-derived fungi display a variety of pharmacological properties, particularly in the field of antitumor and antimicrobial therapy [25].

**Fig. 2:**
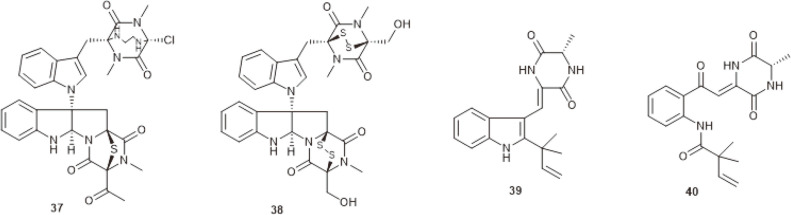
Dioxopiperazines produced by marine-derived *Chaetomium* species.

A new dioxopiperazine alkaloid cristazine (**37**), together with three known ones, chetomin (**38**), neoechinulin A (**39**), and golmaenone (**40**) (Fig. 2), were isolated from the mudﬂat-sediment-derived fungus *C. cristatum*. Compounds **37–40** showed potent radical-scavenging activity against DPPH, with similar IC_50_ values to that of the positive control (ascorbic acid). Compound **37** also displayed cytotoxic activity against human cervical carcinoma (HeLa) cells, with an IC_50_ value of 0.5 μM [26].

Recently, cristazine (**37**) was evidenced to have great potential for inducing apoptosis via the death receptor pathway in human epidermoid carcinoma (A431) cells [27]. Chetomin (**38**) is an antibiotic discovered more than 70 years ago [28]. Recently, it was found to be a potent HIF-1 inhibitor [29] and exhibited antitumor activity in lung cancer, multiple myeloma, and breast cancer [30-32]. Neoechinulin A (**39**) was shown to possess a variety of activities, including anti-inflammatory [33], SARS-CoV-2 M^pro^ inhibiting [34], cytoprotective [35], memory improvement and antidepressant-like effects [36]. In addition, the structure-activity relationship of neoechinulin A revealed that the presence of a diketopiperazine ring was essential for its antioxidant and anti-nitration activities [37].

## Indole Alkaloids

**Fig. 3:**
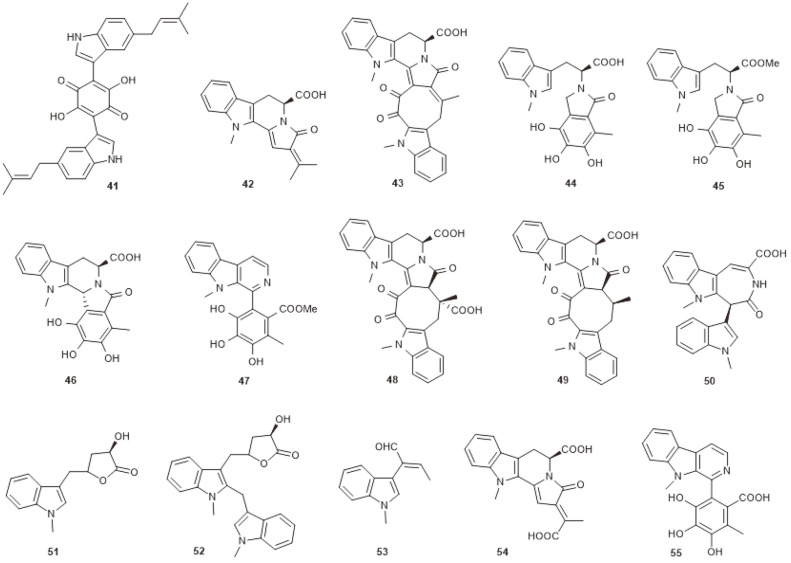
Indole alkaloids produced by marine-derived *Chaetomium* species.

Indole alkaloids are the active moiety of several clinical drugs, such as reserpine, tadalafil and fluvastatin, which are all designed based on an indole skeleton [38, 39]. The indole framework is widely distributed in many fungal natural products [40, 41]. Fig. 3 presents the structures of indole alkaloids produced by marine *Chaetomium*.

Silent fungal Pictet–Spenglerase (FPS) gene activation by 1-methyl-L-tryptophan (1-MT), eight “unnatural” natural indole alkaloids, chaetoglines A−H (**42−49**), were produced by fish-derived *C. globosum* 1C51. Compounds **43** and **47** showed potent antibacterial activity against clinic, pathogenic anaerobes with MIC values ranging from 0.24 to 0.66 μM, which were quite comparable to that of the prescribed antibacterial drug tinidazole. Moreover, compound **47** exhibited moderate AChE inhibitory activity with an IC_50_ value of 4.13 μM [42]. Subsequently, biotransformation by *C. globosum* 1C51, six new indole alkaloids, chaetoindolone A−D (**50−53**), 19-O-demethylchaetogline A (**54**) and 20-O-demethylchaetogline F (**55**), together with two known ones, **42** and **47**, were produced. Compound **50** was found to be antibacterial against *Xanthomonas oryzae* pv. *oryzae* (*xoo*), a pathogen causing rice bacterial leaf blight. Compound **42** was shown to be inhibitory against rape pathogenic fungus Sclerotinia sclerotiorum [43].

## Azaphilones

**Fig. 4:**
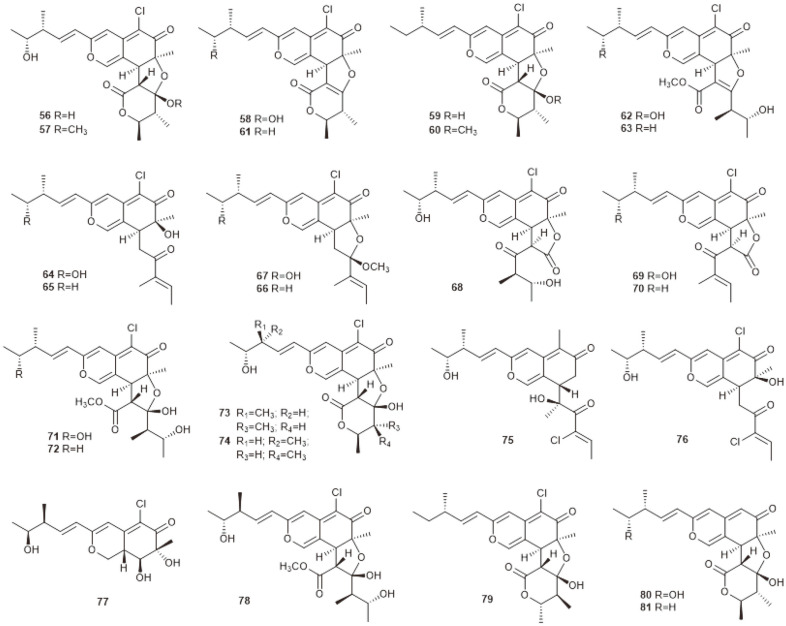
Azaphilones produced by marine-derived *C. globosum* OUPS-T106B-6.

Azaphilones are natural products characterized by an oxygenated bicyclic core that bears an oxygenated nonprotonated carbon in position 7, and are widely distributed in fungi [44, 45]. Azaphilones exhibit broad-spectrum biological activities, including anticancer, antioxidant, anti-inflammatory, antibacterial, antifungal and other activities [46]. Chemical investigation of *C. globosum* OUPS-T106B-6, which was originally isolated from the marine ﬁsh *Mugil cephalus*, resulted in a series of azaphilones being obtained. The compounds included chaetomugilins A–C (**56–58**) [47], D–F (**59–61**) [48], G, H (**62, 63**) [49], I–O (**64–70**) [50], seco-chaetomugilins A, D (**71, 72**) [51], 11-epichaetomugilin A (**73**) and 4’-epichaetomugilin A (**74**) [52], chaetomugilins P–R (**75–77**), 11-epi-chaetomugilin I (**78**) [53], and chaetomugilins S–U (**79–81**) [54] (Fig. 4). All of these were tested for cytotoxicity against human cancer cell lines, and chaetomugilins A, C, F, and I showed signiﬁcant cytotoxic activity against 39 cell lines, while other chaetomugilins exhibited selective growth inhibition of some cultured cancer cell lines. Particularly, chaetomugilin J inhibited PINK1/Parkin-mediated mitophagy to enhance apoptosis in A2780 cells induced by cisplatin [55].

**Fig. 5:**
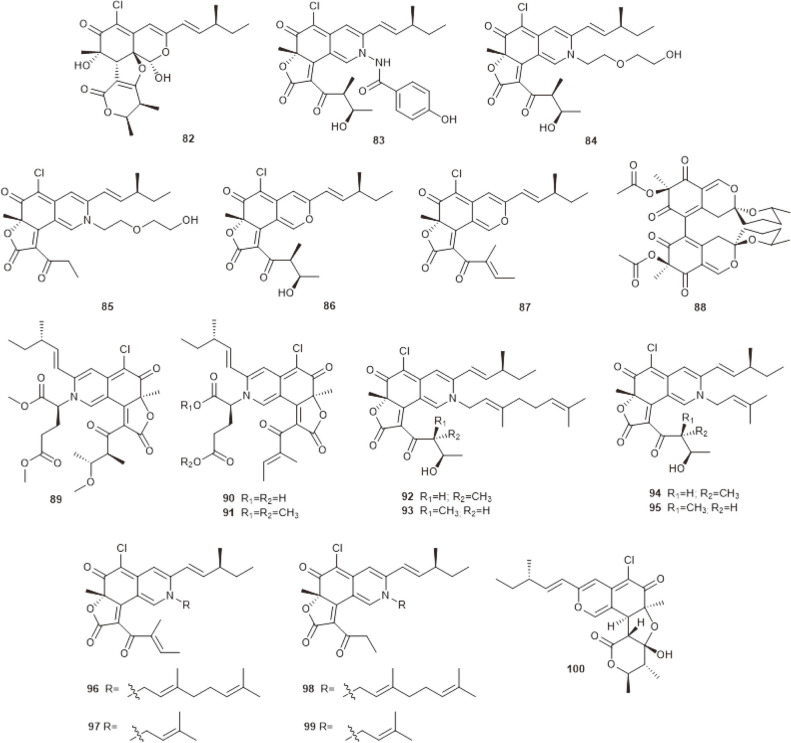
Azaphilones produced by marine-derived *Chaetomium* species.

From the deep-sea-derived fungus *Chaetomium* sp. NA-S01-R1, four novel compounds, chaephilone C (**82**) and chaetoviridides A–C (**83–85**), together with four known compounds, chaetoviridin A (**86**), chaetoviridin E (**87**), chaetomugilin D (**59**) and cochliodone A (**88**), were obtained (Fig. 5). Compounds **82–85** exhibited antibacterial activities against *Vibrio rotiferianus*, *V. vulniﬁcus* or MRSA. Compounds **82–84** also showed strong cytotoxic activities towards the Hep G2 cell or the HeLa cell lines [56]. Three new azaphilones containing glutamine residues, namely N-glutarylchaetoviridins A–C (**89–91**), together with two related compounds, chaetomugilins A and C (**56** and **58**), were isolated from the extract of deep-sea-derived *C. globosum* HDN151398 (Fig. 5). Compounds **91, 56**, and **58** displayed signiﬁcant cytotoxic activity against a broad spectrum of cancer cell lines [57]. Chemical investigation of the deep-sea-derived fungus *C. globosum* MP4-S01-7 led to the discovery of eight new nitrogenated azaphilones (**92−99**) and two known compounds, **86** and **87** (Fig. 5). Most of the compounds exhibited cytotoxicity against the gastric cancer cell lines MGC803 and AGS. Among them, compounds **92, 93**, and **96** exerted the most potent activities, with IC_50_ values less than 1 μM [58].

Except for the antimicrobial and cytotoxic activities mentioned above, chaetomugilins and the closely related chaetoviridins also displayed many other bioactivities. Chaetomugilins A (**56**), D (**59**), I (**64**), J (**65**), O (**70**), Q (**76**) and S (**79**) were reported to have phytotoxic activity [59, 60]. Compounds **56, 59** and **70** exhibited higher activity than **65** and **76**, which could be attributed to the existence of a tetrahydrofuran moiety. Moreover, **70** showed more powerful activity than **56, 59** and **79**, suggesting that the lactone rings may reduce the phytotoxic effects [60]. The discovery indicated that chaetomugilins could be utilized for developing natural eco-friendly herbicides. Chaetomugilins I (**64**) and 11-epi-chaetomugilin I (**78**) were reported to have anti-inflammatory activity. They could remarkably suppress TNF-induced NF-κB activity with an IC_50_ value of 0.9 μM [61]. Chaetoviridin E (**87**) was demonstrated as showing antimalarial activity against *Plasmodium falciparum* with an IC_50_ value of 2.9 μg/ml [62]. And finally, chaetoviridin A (**86**) was indicated to have noticeable antioxidant potential on TLC [63].

## Xanthone Derivatives

**Fig. 6:**
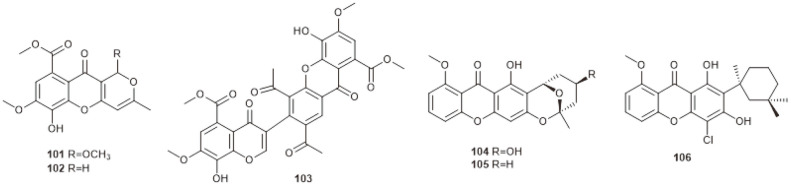
Xanthone derivatives produced by marine-derived *Chaetomium* species.

Xanthones are a class of oxygen-heterocycles containing a γ-pyrone moiety with two aromatic rings. This family of compounds has shown a variety of biological activities, such as α-glucosidase inhibitory activity, antimicrobial activity, anti-inﬂammatory activity and cytotoxicity [64-69]. Xanthones derived from marine *Chaetomium* species are shown in Fig. 6.

Three new natural xanthone derivatives, chaetocyclinone A–C (**101–103**), were produced by the marine algae-derived *Chaetomium* sp. Gö 100/2. Among them, chaetocyclinone A (**101**) exhibited inhibitory activity against phytopathogenic fungi Phytophthora infestans [70]. Investigations of the marine fungus *Chaetomium* sp. 620/GrK 1a led to the discovery of three new fungal polyketide metabolites, chaetoxanthones A–C (**104–106**). Their antiparasitic activity was tested, and compound **105** showed selective activity against *Plasmodium falciparum*, while compound **106** displayed a moderate activity against Trypanosoma cruzi [71].

## Others

**Fig. 7:**
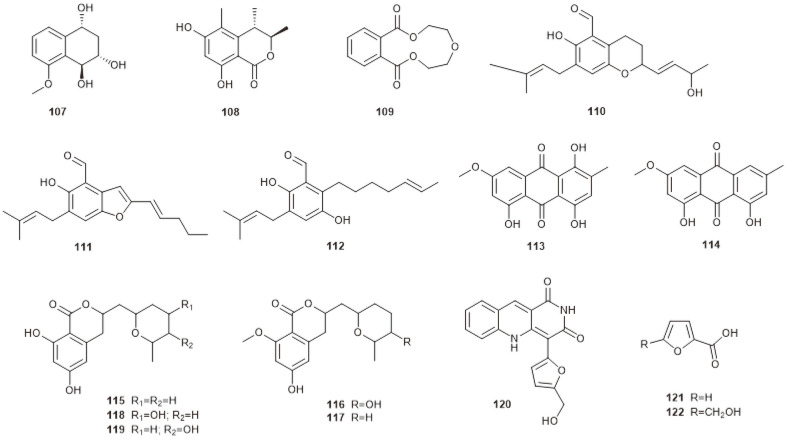
Other compounds produced by marine-derived *Chaetomium* species.

Cultivation of the marine red alga-derived fungus *C. globosum* revealed a new benzaldehyde secondary metabolite chaetopyranin (**110**), together with ten known compounds, including two benzaldehyde congeners, 2-(2’,3-epoxy-1’,3’-heptadienyl)-6-hydroxy-5-(3-methyl-2-butenyl)benzaldehyde (**111**) and isotetrahydroauroglaucin (**112**), two anthraquinone derivatives, erythroglaucin (**113**) and parietin (**114**), five asperentin derivatives including asperentin (**115**), 5’-hydroxy-asperentin-8-methylether (**116**), asperentin-8-methylether (**117**), 4’-hydroxyasperentin (**118**) and 5’-hydroxyasperentin (**119**) (Fig. 7). Compounds **110–113** were evidenced to have moderate DPPH radical-scavenging properties. Compound **110** was also found to have cytotoxicity toward several tumor cell lines [72]. Successive fractionation of the marine fungal *Chaetomium* sp. resulted in obtaining a novel benzonaphthyridinedione derivative, chaetominedione (**120**), and two known fungal metabolites, 2-furancarboxylic acid (**121**) and 5-(hydroxymethyl)-2-furancarboxylic acid (**122**) (Fig. 7). Compounds **120** and **122** showed signiﬁcant inhibitory activity toward TK p56lck tyrosine kinase [73].

## Conclusion

This review summarized 122 secondary metabolites with potent bioactivities derived from marine environment species, reported from 2001 to 2021, and which will benefit future drug development and innovation. As for the structure types of the compounds, we covered cytochalasans (29.51%), dioxopiperazines (3.28%), indole alkaloids (12.30%), azaphilones (36.89%), xanthone derivatives (4.92%) and others (13.11%), indicating the chemical diversity of marine *Chaetomium*. The natural products originating from marine-derived *Chaetomium* species also showed a multiplicity of biological activity, including cytotoxicity, enzyme inhibitory activity, radical-scavenging activity, antiparasitic, antibacterial, and antifungal activity.

It is noteworthy that most of the metabolites were isolated from *C. globosum* strains. When compared to the large number of species (more than 100) contained in *Chaetomium*, very few have been screened for the production of interesting secondary metabolites. This situation may be attributed to the difficulty in cultivating marine microorganisms, especially certain deep-sea-derived fungi that cannot survive under normal laboratory conditions and therefore must be cultured using nontraditional techniques [74, 75]. As a result, the potential of *Chaetomium* genus derived from marine *Chaetomium* remains virtually untapped.

## Figures and Tables

**Table 1:** Natural products isolated from marine-derived *Chaetomium* species.

Structure	Natural product	Species	Bioactivity	Source	Ref.
Cytochalasan	Cytoglobosins A–G (**1–7**) Isochaetoglobosin D (**8**) Chaetoglobosin F_ex_ (**9**)	*C. globosum* QEN-14	Cytotoxicity	Marine green alga *Ulva pertusa*	[13]
Cytochalasan	Chaetoglobosins A, B, J (**10–12**) Halogenated derivatives (**13–21**)	*C. globosum* 1C51	Immunosuppressive activity	Ocean fish *Epinephelus drummondhayi*	[14]
Cytochalasan	Cytoglobosins B (**2**), C (**3**), H (**22**), I (**23**), Chaetoglobosins F_ex_ (**9**), F (**24**), E (**25**), B (**11**), Isochaetoglobosin D (**8**)	*C. globosum* MCCC 3A00607	Antiproliferative activity	Deep-sea sediments	[15]
Cytochalasan	Chaetoglobosins A (**10**), B (**11**), D–F (**26, 25, 24**) Cytoglobosin C (**3**)	*C. globosum* RA07-3	Antibacterial activity	Gorgonian *Anthogorgia ochracea*	[16]
Cytochalasan	Cytoglobosin X (**27**), Chaetoglobosin F_ex_ (**9**), G (**28**), B (**11**)	*C. globosum* E-C-2	Antibacterial activity	Sea cucumber *Apostichopus japonicus*	[17]
Cytochalasan	6-O-methyl-chaetoglobosin Q (**29**) Chaetoglobosins A (**11**), B (**12**), C(**30**), D (**26**), E (**25**), F (**24**), G (**28**), F_ex_ (**9**), V_b_ (**35**), Y (**36**) Aureochaetoglobosin (**31**) Isochaetoglobosin D (**8**) Penochalasin G (**32**) Armochaetoglobin G (**33**) Prochaetoglobosin I (**34**)	*C. globosum* C2F17	Cytotoxicity	Coral *Pocillopora damicornis*	[18]
Dioxopiperazine	Cristazine (**37**) Chetomin (**38**) Neoechinulin A (**39**) Golmaenone (**40**)	*C. cristatum*	Radical-scavenging activity Cytotoxicity Antimicrobial activity Anti-inflammatory effect SARS-CoV-2 Mpro Inhibitor Cytoprotection Memory improvement Antidepressant-like effects	Mudflat sediment	[26]
Indole alkaloid	Cochliodinol (**41**)	*C. globosum* E-C-2	None	Sea cucumber *Apostichopus japonicus*	[17]
Indole alkaloid	Chaetogline A−H (**42−49**) Chaetoindolone A−D (**50−53**) 19-O-Demethylchaetogline A (**54**) 20-O-Demethylchaetogline F (**55**)	*C. globosum* 1C51	Antibacterial and antifungal activity	Marine fish *Epinephelus drummondhayi*	[42, 43]
Azaphilone	Chaetomugilins A–O (**56–70**) Seco-chaetomugilins A, D (**71, 72**) 11-Epichaetomugilin A (**73**) 4’-Epichaetomugilin A (**74**) Chaetomugilins P–R (**75–77**) 11-epi-Chaetomugilin I (**78**) Chaetomugilin S–U (**79–81**)	*C. globosum* OUPS-T106B-6	Cytotoxicity	Marine fish *Mugil cephalus*	[47-54]
Azaphilone	Chaephilone C (**82**) Chaetoviridides A–C (**83–85**) Chaetoviridins A, E (**86, 87**) Chaetomugilin D (**59**) Cochliodone A (**88**)	*Chaetomium* sp. NA-S01-R1	Antibacterial activity and cytotoxicity	Deep sea water	[56]
Azaphilone	N-glutarylchaetoviridins A–C (**89–91**) Chaetomugilins A, C (**56,58**)	*C. globosum* HDN151398	Cytotoxicity	Deep sea sediment	[57]
Azaphilone	Nitrogenated azaphilones (**92−99**) Chaetoviridins A, E (**86, 87**)		Cytotoxicity	Deep sea water	[58]
Azaphilone	Chaetoviridins A (**86**), E (**87**), B (**100**) Chaetomugilin A (**56**)	*C. globosum* E-C-2	None	Sea cucumber	[17]
Xanthone derivative	Chaetocyclinone A–C (**101–103**)	*Chaetomium* sp. Gö 100/2	Antifungal activity	Marine algae	[70]
Xanthone derivative	Chaetoxanthones A–C (**104–106**)	*Chaetomium* sp. 620/GrK 1a	Anti-parasitic activity	Marine algae	[71]
Tetralone derivative	Xylariol A (**107**)	*C. globosum* RA07-3	None	Gorgonian *Anthogorgia ochracea*	[16]
Isocoumarin derivative	(3R,4S)-6,8-Dihydroxy-3,4,5-trimethylisochroman-1-one (**1**08)	*C. globosum* RA07-3	None	Gorgonian *Anthogorgia ochracea*	[16]
O-phthalate derivative	2,5,8-Benzotrioxacycloundecin-1,9-dione (**109**)	*C. globosum* RA07-3	None	Gorgonian *Anthogorgia ochracea*	[16]
Benzaldehyde derivative	Chaetopyranin (**110**) 2-(2’,3-Epoxy-1’,3’-heptadienyl)-6-hydroxy-5-(3-methyl-2-butenyl)benzaldehyde (**111**) Isotetrahydroauroglaucin (**112**),	*C. globosum* CCTCC AF 206003	Radical-scavenging activity Cytotoxicity	Marine red alga *Polysiphonia urceolata*	[72]
Anthraquinone derivative	Erythroglaucin (**113**) Parietin (**114**)	*C. globosum* CCTCC AF 206003	Radical-scavenging activity	Marine red alga *Polysiphonia urceolata*	[72]
Asperentin derivative	Asperentin (**115**) 5’-Hydroxy-asperentin-8-methylether (**116**) Asperentin-8-methyl ether (**117**) 4’-Hydroxyasperentin (**118**) 5’-Hydroxyasperentin (**119**)	*C. globosum* CCTCC AF 206003	None	Marine red alga *Polysiphonia urceolata*	[72]
Benzonaphthyrid inedione derivative	Chaetominedione (**120**)	*Chaetomium* sp.	Enzyme inhibitory activity	Marine alga *Valomia utricularis*	[73]
Furan derivatives	2-Furancarboxylic acid (**121**) 5-(hydroxymethyl)-2-Furancarboxylic acid (**122**)	*Chaetomium* sp.	Enzyme inhibitory activity	Marine alga *Valomia utricularis*	[73]
